# Transpedal and Tibiopedal Retrograde Revascularization for Peripheral Vascular Disease

**DOI:** 10.7759/cureus.22082

**Published:** 2022-02-10

**Authors:** Nawaf M O S Ali, Mohammad H A A A Alsaffar

**Affiliations:** 1 Vascular Surgery Unit, Department of Surgery, Jaber Al-Ahmad Hospital, Mubarak Al-Kabeer Health Region, KWT

**Keywords:** peripheral vascular surgery, angioplasty, angiography, transpedal, tibiopedal

## Abstract

Peripheral vascular disease, or peripheral artery disease (PAD), is a chronic and debilitating disease that affects millions of people worldwide. PAD is associated with abnormal arterial narrowing, specifically outside of the heart and brain. PAD is primarily observed in the legs, but it can also affect the kidneys, arms, and neck. Patients with PAD often complain of acute leg pain that occurs when walking. However, the pain resolves with rest. The phenomenon of acute pain due to narrowed arteries is known as intermittent claudication. Common symptoms of PAD include abnormal hair and nail growth, bluish skin, skin ulcers, and cold skin. Untreated and unmanaged PAD can lead to serious complications such as tissue infection or necrosis, which in turn could lead to amputation. In rare cases, PAD may cause a stroke or coronary artery disease.

Among all the management options available, the endovascular approach remains the recommended and the gold standard nowadays. In this paper, we examine and analyze the transpedal and tibiopedal retrograde revascularization in PAD patients in which the conventional antegrade approach is not successful intra-operatively with emphasis on the challenges and postoperative complications. It also correlates the different studies and its outcomes with an up-to-date worldwide results.

## Introduction and background

An estimated 200 million people worldwide are affected by peripheral artery disease (PAD), though most of them remain asymptomatic [1]. The prevalence of PAD is low among the younger population, and the rate increases with age. An estimated 20% of elderly people (>80 years) suffer from PAD. In terms of ethnicity, African Americans are twice as likely to be affected by the disease as non-Hispanic Whites, with age being a major factor [1]. Among PAD patients who experience symptoms, 80% to 90% have problems with the popliteal and femoral arteries. Moreover, approximately 3% to 4% of patients with PAD undergo amputation. Patients with the disease are also at high risk of heart failure [1]. As per the American Heart Association (AHA), some of the risk factors of PAD include the following: (a) increased age; (b) high cholesterol and high blood pressure; (c) a history of smoking; (d) and pre-existing metabolic disorders, primarily diabetes mellitus [2]. PAD is associated with significant morbidity and mortality, irrespective of symptoms [3]. It is a major health concern that has received relatively little public attention and research. After stroke and coronary artery disease, PAD is the third-most common issue associated with atherosclerosis [3].

## Review

Endovascular interventions for PAD

The past few decades have witnessed significant improvements in endovascular therapies. The treatment and management of PAD percutaneously is the current trend among surgeons and clinicians. In patients with multiple comorbidities, less invasive endovascular interventions are highly recommended. Endovascular treatments are a safe and an effective alternative to open surgeries [4]. Endovascular interventions include arterial imaging and non-invasive physiological tests. The process is well defined and helps surgeons localize the lesion or disease and plan the procedure accordingly. The indication and timing for revascularization are based on three primary clinical presentations: acute limb ischemia, critical limb ischemia (CLI), and presentation of claudication [4]. Medical treatments and physical therapies are often the first-line approach for patients with claudication. However, when first-line treatments fail to improve function and quality of life, endovascular interventions are considered. In cases of acute limb ischemia and CLI, endovascular interventions are considered a necessity, and urgent revascularization is required [4].

The tibiopedal approach (TPA)

Treating and managing endovascular disease and associated complications are often complex and challenging tasks. Conventional endovascular approaches have many limitations, such as bleeding complications, lack of adequate vascular access, and excess contrast use-induced renal impairment. In recent years, the tibiopedal arterial minimally invasive retrograde revascularization (TAMI) approach has been found to mitigate the risk of conventional endovascular therapy. TAMI is highly recommended for patients who are not eligible for antegrade common femoral artery (CFA) access, conventional retrograde CFA access, or vascular bypass surgery, because of its overall safety and effectiveness.

The TAMI technique is based on the use of single transtibial artery access with the help of a micro-puncture access kit. During the procedure, the surgeon uses a transpedal sheath. The TAMI technique involves a retrograde approach via the tibiopedal access. Intra-arterial vasodilators are required for access preservation as well as for the prevention of thrombosis and vasospasm. The TAMI technique ensures that the activated clotting time remains within the 250- to 300-second range. Radical compression devices or manual compression are often used to achieve hemostasis [5].

In a recent multicenter observational study, researchers evaluated the safety and efficacy of tibiopedal access for patients with severe lower-extremity ischemia. Among the patients in the study, the success rate of technical tibiopedal access was high (93.4%) compared to that of the technical occlusion crossing technique (85.3%). All patients had successful tibial access. Tibiopedal access was associated with significant improvement in patients who underwent a crossing of infrainguinal lesions for severe lower-limb ischemia [6].

A recent prospective interventional study reported the success of popliteal and tibiopedal retrograde revascularization. Researchers indicated that patients with infrainguinal lesions could be treated successfully with popliteal and tibiopedal retrograde access as a rescue intervention. Per the report, tibiopedal retrograde access is an effective alternative endovascular approach with low 30-day mortality and morbidity rates compared to conventional endovascular approaches [7]. The demand for retrograde tibiopedal access has increased as a salvage approach for cases where approach via lesion from antegrade is not possible. The TPA has been widely used for total occlusions of infrainguinal arteries as a standard bailout revascularization approach. In conventional methods, below-the-knee (BTK) chronic occlusions are difficult to treat and come with several complications [8].

**Figure 1:**
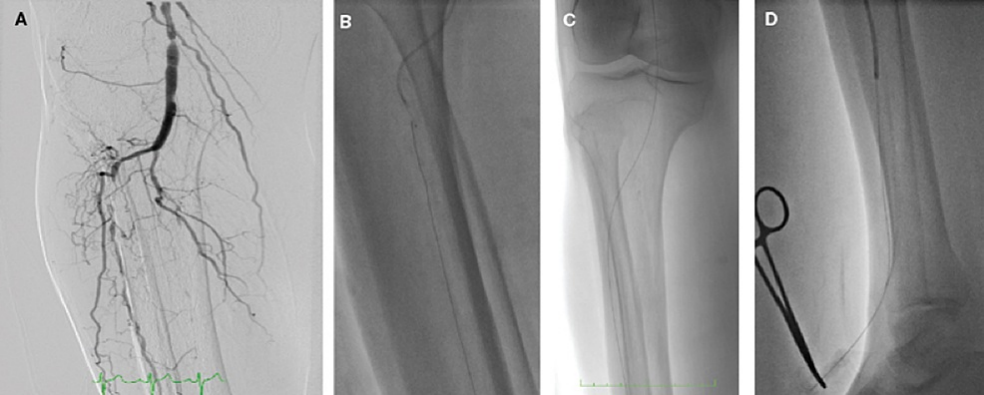
The successful crossing of the anterior tibial artery occlusion. (A) Occlusion found in the anterior tibial artery. (B) Use of microsnare for snaring of the 0.018-in wire (V-18TM). (C) Wire inserted within the occluded segment. (D) The distal end of the wire.

The tibiopedal bypass approach has been effective in treating severe limb issues in diabetic patients as well. In a recent report, a 63-year-old patient suffered from ischemic heel ulcer with intermittent claudication. The oscalcis was left exposed due to infection. A combination of tibiopedal bypass grafting and free tissue transfer was used as salvage for limb preservation. The procedure resulted in rapid wound healing, and the patient started walking by day 26. Thus, tibiopedal bypass grafting can be used in selective cases as a salvage limb revascularization technique [9]. In conventional settings, an antegrade or retrograde femoral artery approach is required for tibial lesions. In approximately 20% of patients, a crossover of the lesion was not permitted. However, in the early 1990s, researchers demonstrated that the retrograde pedal/tibial approach was useful in cases where conventional crossing over of the tibial lesion had failed. A classic example of occlusion crossover with the help of snaring using a microsnare was demonstrated by El-Sayed [10]. The microsnare was inserted with the guide wire from the CFA access (Figure 1).

Patients with femoropopliteal (FP) chronic total occlusion (CTO) are often difficult to treat. Conventional surgical approaches are often associated with acute limb ischemia, access site bleeding, worsening kidney dysfunction, and hematoma within a 30-day postoperative timeframe [11].

In a recent study comprising 123 patients, Htun et al. [11] reported the clinical efficacy and safety of the primary retrograde TPA in the treatment of FP-CTO. None of the patients reported any of the acute complications associated with conventional surgical approaches. In a recent review, researchers claimed the superiority of TPA for lower-limb arterial occlusion. The TPA is associated with minimal complications and positive clinical outcomes compared to conventional endovascular intervention [12].

**Figure 2:**
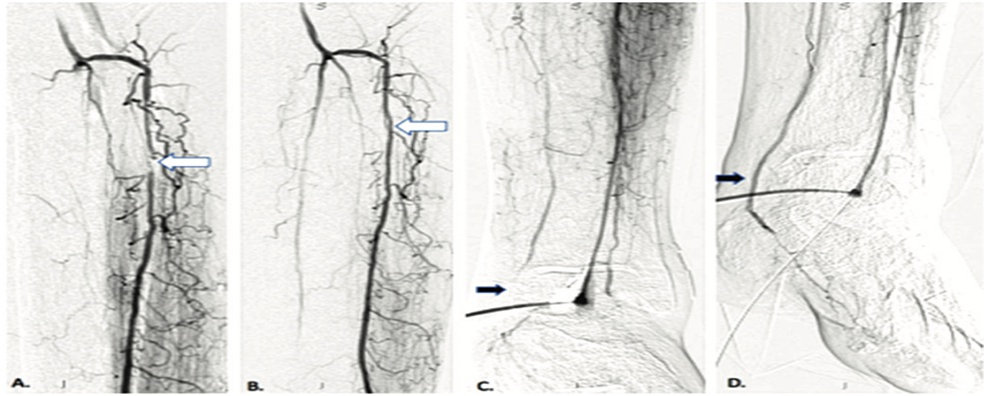
Crossover in the anterior tibial artery with the help of retrograde revascularization of the posterior tibial. (A) The artery lesion (white arrow). (B) Revascularized artery (white arrow). (C) Completely occluded posterior tibial artery (black arrow). (D) Revascularized posterior tibial artery (black arrow).

The TPA has emerged as an effective treatment for revascularization of BTK lesions. In an observational cohort of 194 patients, researchers observed no differences between patients who underwent TPA with transradial guidance and those who underwent TPA without it. Crossover in the anterior tibial (AT) artery with the help of retrograde revascularization of the posterior tibial can be viewed in Figure 2 [13]. The treatment of chronic limb ischemia and BTK lesions using the TPA approach was safe and feasible. The process was associated with low access-site complications and a high success rate [13].

The transpedal approach

Patients with CLI often require a multidisciplinary approach. The treatment of CLI comprises revascularization procedures and medical therapy. Both procedures are required for a faster recovery and a better quality of life. Conventional surgical procedures are unfeasible and complex if the affected lesion is found below the knee as it is usually associated with acute postsurgical complications. Thus, unique limb revascularization procedures such as transpedal angiography have been found to be safe, effective, and feasible for patients with CLI [14].

In the past decade, atherosclerotic disease of the superficial femoral artery (SFA) has been commonly found in adults. Percutaneous interventions have been the mainstay treatment approach for SFA, primarily via femoral artery access. However, there is limited research on or evidence for the transpedal artery access as an intervention for SFA. A routine primary transpedal artery approach for patients who require SFA stenting has not been documented. In a first-of-its-kind study, researchers examined the impact of the transpedal approach, guided by an ultra-low profile 6 Fr sheath, on SFA. Six patients received unique treatment. The researchers found the following: (i) Pedal artery can be used as a single-access site for successful STA stenting with the help of 6 Fr Slender sheath. (ii) None of the patients required contralateral femoral artery access. (iii) No acute pedal artery access-site complication was observed in the 30-day postoperative period. (iv) A duplex ultrasound on a 1-month follow-up revealed 100% access-site recovery [15]. Thus, the researchers claimed the first successful SFA intervention a single transpedal approach that required a stent placement for lower-limb angiography.

Based on the current evidence, there are several options to treat occluded bypass grafts. These include catheter-based thrombolysis, orthrombectomy, balloon angioplasty, and repeat bypass grafting. Occluded bypass grafts can be treated with surgical revision (current gold standard treatment). Surgical revision is known to have superior patency over conventional endovascular treatment. However, surgical revisions are associated with several complications and limitations such as long procedure time, high rates of nerve injury, bleeding, long duration of hospitalization, wound and graft infection, and increased pain. Thus, endovascular approaches, such as transpedal angiography, are considered safer. Transpedal angiography is associated with low complications and better health outcomes.

In a recently published care report, a team of surgeons performed revision surgery of the fem-pop artery with transpedal approach. The surgeons used percutaneous angioplasty and stenting to perform the surgery. It is a unique case that emphasizes on the need for percutaneous intervention such as transpedal pathway for hard-to-treat occluded artery lesions [16]. A combination of retrograde and antegrade transpedal approach was useful in treating or recanalizing infrainguinal arteries. However, the challenge of such approaches arises when patients present with popliteal or infrapopliteal arteries [17]. In such complex cases, retrograde access is not possible, and, hence, transpedal approach is unsuitable.

Advantages of the transpedal and tibiopedal endovascular approach

One of the main advantages of endovascular treatment is minimal access to the arterial site. In the context of transpedal approach, a low rate of artery access-site complications is considered the main advantage over conventional surgical approaches. However, there are several other advantages of endovascular treatments [15]. Transpedal access is a simple and straightforward approach for microvascular complications. For example, it prevents the need for crossing over. The approach is simple and takes less time compared to conventional open surgery techniques. Another vital approach is ipsilateral antegrade that is known to have similar efficacy and safety as the transpedal access approach [15].

In complex cases of aortic bifurcations, the risk of radiation exposure and procedure time increase. A simple transpedal approach to revise aortic bifurcations can prevent the need for higher radiation doses and reduce the operating time. The transpedal approach is helpful for both patients and surgeons [15]. There is strong evidence that it reduces radiation exposure (less scattered radiation) to the lower mass content as compared to the abdominal region. Low radiation exposure is one of the main advantages of endovascular treatments. Transpedal and tibiopedal approaches for vascular diseases are associated with reduced post-surgical ambulation. The recovery time is increased while patient satisfaction is said to improve. A lower ambulation time is directly linked with higher workflow efficiency [15].

There is no evidence for the value or economic impact of adopting transpedal or tibiopedal approaches for vascular diseases [15]. However, researchers have stated that fast recovery rates and low operating times could translate to low staffing needs and low demand facility resources. The recent development in endovascular treatment has increased the demand for transpedal tibiopedal approaches. Patients who were previously denied or excluded due to femoral access difficulties can now be treated and managed successfully with endovascular treatment [15].

Disadvantages of the transpedal and tibiopedal endovascular approach

Although transpedal and tibiopedal endovascular treatments are better than known conventional surgical approaches, they have several limitations. In patients with total occlusion of the SFA, the transpedal approach can render useless. It is unlikely for the surgeon to define the extent of the lesion [15]. Thus, the transpedal approach could be a challenge in such complex cases. Many surgeons find it difficult even after obtaining access to the proximal area of the vessel-occluded distal AT artery post-DP access. The process is highly complex and challenging. There are several clinical problems that surgeons must overcome before angiography [15].

In critical patients, alternative access can be provided. However, the benefits should outweigh the risks. In cases where transpedal and tibiopedal endovascular approaches fail, surgeons would prefer the conventional femoral artery puncture approach. Transpedal approach is beneficial for patients with a limited or single tibial vessel. The main limitation is the inability to support multiple tibial vessels [15]. Although the intervention can be performed in retrograde form, the presence of multiple angulated vessel bifurcations makes the process challenging to complete. Surgeons would find it difficult to make the guidewire manipulation. In most cases, surgeons can opt for a conventional femoral approach to treat multiple vessel issues. Thus, the transpedal and tibiopedal endovascular approaches may have several limitations, and the treatment should be applied based on individual case scenarios [15].

## Conclusions

PAD is a major health concern globally and its prevalence has notably increased over the past years. Regardless of symptoms, it is associated with significant morbidity and mortality. Contributors that may put people at high risk of developing PAD include, but not limited to, hypertension, diabetes, and dyslipidemia. Percutaneous vascular intervention for acute or chronic peripheral arterial disease is the current trend, especially in patients with multiple comorbidities, as it is a safe and an effective alternative to open surgery. Due to its long-term durability and limited complications, endovascular treatment is by far the most recommended intervention. Surgeons should assess the risk of such procedures and provide their patients with individualized plans based on the current updated studies and promising results mentioned earlier in this paper.
